# Supplementary material for: Risk of Severe COVID-19-Related Outcomes among Patients with Cirrhosis: A Population-Based Cohort Study in Canada
Source: Viruses. 2024 Feb 24;16(3):351. doi: 10.3390/v16030351 (PMC10975436; doi:10.3390/v16030351)
Supplement: Supplementary file 1 [file viruses-16-00351-s001.zip › viruses-2843200-supplementary.pdf]

Supplementary Data

Risk of Severe COVID-19-Related Outcomes among Patients with Cirrhosis: A Population-Based Cohort Study in Canada

Héctor Alexander Velásquez García [1,2,†], Prince A. Adu [1,3,†,*], Ada Okonkwo-Dappa [4], Jean Damascene Makuza [1,2], Georgine Cua [1,2], Mawuena Binka [2], James Wilton [1], Hind Sbihi [1] and Naveed Z. Janjua [1,2,5,*]

[1] British Columbia Centre for Disease Control, Vancouver, BC V5Z 4R4, Canada; hector.velasquez@bccdc.ca (H.A.V.G.); jean.makuza@bccdc.ca (J.D.M.); georgine.cua@bccdc.ca (G.C.); james.wilton@bccdc.ca (J.W.); hind.sbihi@bccdc.ca (H.S.)
[2] School of Population and Public Health, University of British Columbia, Vancouver, BC V6T 1Z3, Canada; mawuena.binka@bccdc.ca
[3] Department of Social Medicine, Heritage College of Osteopathic Medicine, Ohio University, Dublin, OH 43016, USA
[4] Department of Family Practice, University of British Columbia, Vancouver, BC V6T 1Z4, Canada; ada.okonkwodappa@ubc.ca
[5] Centre for Advancing Health Outcomes, St. Paul's Hospital, Vancouver, BC V6Z 1Y6, Canada
* Correspondence: prince.adu@alumni.ubc.ca (P.A.A.); naveed.janjua@bccdc.ca (N.Z.J.)
† These authors contributed equally to this work.

Section S1. BC COVID-19 Cohort (BCC19C)

The BC COVID-19 Cohort (BCC19C) is a surveillance platform that integrates COVID-19 data with administrative health datasets (Table S1). The platform was established in 2020 in order to support the public health response to the COVID-19 pandemic. The BCC19C is a dynamic cohort in which COVID-19 datasets are updated daily and most administrative health datasets are updated weekly/monthly. The majority of the integrated datasets are population- based (cover all individuals in the province). Datasets are de-identified and linked together through a combination of probabilistic and deterministic matching algorithms, as described previously.[1]

The BCC19C is a collaboration between the Provincial Health Services Authority (PHSA), BC Centre for Disease Control (BCCDC), and the BC Ministry of health (MOH). The platform was established under BCCCDC's public health mandate. This study was also reviewed and approved by the Behavioral Research Ethics Board at the University of British Columbia (H20-02097).

Table S1. Data Sources integrated within the BC COVID-19 Cohort (BCC19C)

**British Columbia Centre for Disease Control (BCCDC), Provincial Heath Services Authority (PHSA) and Regional Health Authority data sources:**	**Data Date Ranges:**
Integrated COVID-19 laboratory dataset (SARS-CoV2 tests from private/public labs) [S1]	Jan,2020-onward
COVID-19 surveillance case data (information collected on all probable/confirmed cases as part of public health follow up) [S2]	Jan,2020-onward
Provincial COVID-19 Monitoring Solution (critical and non-critical care hospital census data) [S3]	Jan,2020-onward
Provincial Immunizations Registry (COVID-19 vaccination data) [S4]	Dec,2020-onward
Provincial Laboratory Information Solution (laboratory tests from private/public labs) [S5]	Jan,2020-onward
Public Health Reporting Data warehouse (Influenza laboratory tests)[S6]	Jan,2008-onward
Emergency department visits (hospital-based and community-based ambulatory care)	Mar,2020-onward
Ministry of Health (MoH) Administrative Data Sources:	**Data Date Ranges:**
Client Roster (CR) (registry of enrollment in the universal public health insurance plan including residential history) [S7]	2008/9-onward
Discharge Abstracts Database (DAD) (hospital discharge records) [S8]	2008/9-onward
Medical Services Plan (MSP) (physician diagnostic and billing data for services provided through universal public health insurance plan) [S9]	2008/9-onward
PharmaNet (Pharma) (prescription drugs dispensed from community pharmacies, includes medications covered by public and private insurance plans) [S10]	2008/9-onward
BC Vital Statistics (VS) (deaths registry) [S11]	2008/9-onward
National Ambulatory Care Reporting System (NACRS) (hospital-based and community- based ambulatory care) [S12]	2011/12–onward
Chronic Disease Registry [S13]	2008/9-2018/19
811 Calls (respiratory calls only) [S14]	2014-onward
Health System Matrix [S15]	2018/19-onward

References (Data Sources)

S1. British Columbia Centre for Disease Control [creator]. Integrated COVID-19 laboratory dataset (SARS-CoV2 tests from private/public labs). Public Health Reporting Data Warehouse, British Columbia Centre for Disease Control [publisher] (2020). 2021.

S2. British Columbia Centre for Disease Control [creator]. COVID-19 surveillance case data. British Columbia Centre for Disease Control [publisher]. (2020). 2021.

S3. Provincial Health Services Authority [creator]. Provincial COVID-19 Monitoring Solution. Provincial Health Services Authority [publisher]. (2020). 2021.

S4. Provincial Health Services Authority [creator]. Provincial Public Health Information Systems [publisher]. (2020). 2021.

S5. Provincial Health Services Authority [creator]. COVID-19 vaccination data. Provincial Immunizations Registry, Provincial Public Health Information Systems [publisher]. (2020). 2021.

S6. British Columbia Centre for Disease Control [creator]. Respiratory datamart, Public Health Reporting Data Warehouse, British Columbia Centre for Disease Control [publisher] (2020). 2021.

S7. British Columbia Ministry of Health [creator]. Client Roster (Client Registry System/Enterprise Master Patient Index). British Columbia Ministry of Health [publisher]. Data Extract. MOH (2020). 2021. https://www2.gov.bc.ca/gov/content/health/health-forms/online-services

S8. British Columbia Ministry of Health [creator]. Discharge Abstract Database (Hospital Separations). British Columbia Ministry of Health [publisher]. Data Extract. MOH (2020). 2021. https://www2.gov.bc.ca/gov/content/health/health-forms/online-services

S9. British Columbia Ministry of Health [creator]. Medical Services Plan (MSP) Payment Information File. British Columbia Ministry of Health [publisher]. Data Extract. MOH (2020). 2021.https://www2.gov.bc.ca/gov/content/health/health-forms/online-services

S10. British Columbia Ministry of Health [creator]. PharmaNet. British Columbia Ministry of Health [publisher]. Data Extract. MOH (2020). 2021.https://www2.gov.bc.ca/gov/content/health/health-forms/online-services

S11. BC Vital Statistics Agency [creator]. Vital Statistics Deaths. BC Vital Statistics Agency [publisher]. Data Extract. BC Vital Statistics Agency (2020). 2021. https://www2.gov.bc.ca/gov/content/health/health- forms/online-services

S12. British Columbia Ministry of Health [creator]. National Ambulatory Care Reporting System. British Columbia Ministry of Health [publisher]. Data Extract. MOH (2020). 2021. https://www2.gov.bc.ca/gov/content/health/health-forms/online-services

S13. British Columbia Ministry of Health [creator]. Chronic Disease Registry. British Columbia Ministry of Health [publisher]. Data Extract. MOH (2020). 2020. https://www2.gov.bc.ca/gov/content/health/health-forms/online-services

S14. British Columbia Ministry of Health [creator]. 811 calls. British Columbia Ministry of Health[publisher]. Data Extract. MOH (2020). 2021. https://www2.gov.bc.ca/gov/content/health/health-forms/online-services

S15. British Columbia Ministry of Health [creator]. Health System Matrix. British Columbia Ministry of Health [publisher]. Data Extract. MOH (2020). 2021. https://www2.gov.bc.ca/gov/content/health/health-forms/online-services

S16. British Columbia Ministry of Health [creator]. Population Grouper Methodology. British Columbia Ministry of Health [publisher]. Data Extract. MOH (2020). 2021. https://www2.gov.bc.ca/gov/content/health/health-forms/online-services

Section S2. Definitions for comorbidity variables derived from administrative datasets

Administrative data sources:

A) Chronic disease registry (CDR)[5] – Available information includes diagnosis date(s)
B) Medical Services Plan (MSP)[1] – ICD-9 billing/diagnostic codes.
C) Discharge Abstract Database (DAD)[3] – DAD1 contains ICD-9 coded hospitalization data and DAD2 contains ICD-10 coded hospitalization data.
D) National Ambulatory Care Reporting System (NACRS)[4] – Contains ICD-10 coded diagnostic codes.
E) PharmaNet[2] – Each medication is identified with a drug identification number (DINPIN).
F) Canadian Census (2016)
G) Provincial Immunization Registry

All comorbidities were identified according to the information extracted from CDR, with the following exceptions:

1. Cirrhosis

- MSP ICD-9 diagnostic codes: starting with 4562; exact codes 4560, 4561, 56723, 5722, 5724, 5712, 5728, 5715, 7895, 07044, 5713
- DAD1 ICD-9-CM diagnostic codes: starting with 4562; exact codes 4560, 4561, 56723, 5722, 5723, 5724, 5712, 5728, 5715, 7895, 07044, 5713
- DAD2/NACRS ICD-10-CA diagnostic codes: starting with K703; exact codes: I850, K652, K721, K729, K766, K767, K7460, K7469, R18, I98.20, I98.3, K704

2. Cancer (ever)

Any occurrence of "lymphoma", "metastatic cancer", and "solid tumor without metastasis" groups of Elixhauser comorbidity index[3].

3. Chronic kidney disease

As per "renal failure" group of Elixhauser comorbidity index[3].

4. Diabetes (DM)

Diabetes status as per CDR; classified as:

DM requiring insulin: PharmaNet DINPINs 1934074, 1959220, 2024284, 586714, 2024233, 446564, 612227, 1934112, 612197, 632651, 632686, 1986085, 1986805, 1986813, 2025256, 1959239, 2024268, 587737, 2024225, 446572, 612235, 1934066, 646148, 2024241, 446580, 612278, 1934090, 2024403, 2241310, 2415089, 2403447, 795879, 2024217, 1959212, 2025248, 889121, 2024446, 1962639, 2024292, 1962655, 2024306, 1962663, 2024322, 1962647, 2024314, 889091, 889105, 889113, 2466864, 644358, 733075, 2024276, 513644, 2275872, 514551, 552275, 612170, 1985949, 2022249, 2275864, 514535, 612162, 612359, 2244353, 2245397, 2377209, 2460416, 2460424, 2460408, 2265435, 2265443, 2467879,

2467887, 2474875, 2412829, 2271842, 2245689, 2276410, 2251930, 2444844, 2294338, 2444852, 2461528, 2441829, 2493373, 2478293, 2279479, 2279460, 2279487, 2294346, 2229704, 2469901, 2229705, 2469898, 2233562, 2241283, 2403412, 2469871, 2439611, 2470152, 2240294, 2240295, 2403420, 2240297, 2403439, 632694, 650935, 1986821, 2022230, 552259, 614416, 1985957, 1985981,

612189, 552267, 628301, 723789, 1985930, 446610, 612219, 612200, 446599, 612243, 1934104, 446602, 612251, 1934082, 773654, 1985973, 632678, 1985965, 5894, 6009, 274119, 274127, 275409, 275417, 275425, 539201, 539244, 542911, 542938, 542946, 546348, 554820, 648094, 999717, 1986791, 45230001, 45230002, 45230003, 45230004, 45230005, 45230006, 45230007, 45230008, 45230009, 45230010, 45230011, 45230012, 45230013, 47450001, 47450002, 47450003, 47450004, 47450005, 47450006, 47450007, 66123203, 66124134, 66124135, 66124225, 66124582, 45230014, 45230015, 45230016, 46340034, 46340035, 46340036, 46340037, 47450008, 47450009.

- DM treated without insulin: Any record lacking the aforementioned DINPINs.

5. Immunosuppression

Based on Sundaram et al. 2020[4]. Includes any occurrence in DAD/NACRS of:

- Antibody- or leukocyte-related immune disorders: ICD-10: D80 - D84, D89.8, D89.9 / ICD-9: 279
- Neutropenia - ICD-10: D70 / ICD-9: 288.0
- Abnormality of leukocytes - ICD-10: D71, D72 / ICD-9: 288.1, 288.2
- Splenomegaly - ICD-10: D73.0 - D73.2 / ICD-9: 289.4 or 289.5
- Asplenia - ICD-10: Q890 / ICD-9: 759.0
- Sickle cell syndrome - ICD-10: D570 - D572; D57.8 / ICD-9: 282.6
- Transplant: Organ or tissue replaced by transplant - ICD-9 (MSP): V42; Transplanted organ and tissue status - ICD-10: Z94
- AIDS/HIV – As per "AIDS/HIV" group of Elixhauser comorbidity index[3].

6. Injection Drug Use[5]

Diagnosis age between 11 and 65:

- MSP ICD-9 diagnostic codes: starting with 292, 970, 3040, 3041, 3042, 3044, 3045, 3046, 3047, 3048, 3049, 3054, 3055, 3056, 3057, 3059, 6483, 7960, 9621, 9650, 9658, 9663, 9664, 9670, 9684, 9685, 9694, 9696, 9697, 9698, 9699, E8500, V6542; exact code V6542
- DAD1 ICD-9-CM diagnostic codes: starting with 292, 970, 3040, 3041, 3042, 3044, 3045, 3046, 3047, 3048, 3049, 3054, 3055, 3056, 3057, 3059, 6483, 7960, 9621, 9650, 9658, 9663, 9664, 9670, 9684, 9685, 9694, 9696, 9697, 9698, 9699, E8500, V6542; exact code V6542
- DAD2/NACRS ICD- 10-CA diagnostic codes: starting with F11, F13, F14, F15, F19, R781, R782, T387, T400, T401, T402, T403, T404, T405, T406, T408, T409, T412, T423, T424, T425, T426, T427, T428, T436, T438, T439, T507; exact codes: R781, R782, T387, T400, T401, T402, T403, T404, T405, T406, T408, T409, T412, T423, T424, T425, T426, T427, T428, T436, T438, T439, T507
- NACRS complaint codes: starting with 751, 753

7. Alcohol Misuse[6]

- MSP ICD-9 diagnostic codes: starting with 291, 303, 3050, 3575, 4255
- DAD1 ICD-9-CM: starting with 291, 303, 3050,3575, 4255
- DAD2/NACRS ICD-10-CA: starting with F10, E244, G312, G621, G721, I426, Z502, Z714.

8. Schizophrenia and psychotic disorders[7]

2 MSP occurrences, or 1 DAD, or 1 NACRS

- MSP ICD-9 diagnostic codes: starting with 295, 2988, 2989, 2970, 2972, 2971, 2973, 2978, 2979
- DAD1 ICD-9-CM: starting with 295, 2988, 2989, 2970, 2972, 2971, 2973, 2978, 2979
- DAD2/NACRS ICD-10-CA: starting with F20, F21, F22, F23, F24, F25, F28, F29.

9. Intellectual and developmental disabilities

- ICD-9

- 317 = Mild Mental Retardation (MR).
- 318 = Moderate, severe, and profound MR.
- 319 = Unspecified MR.
- 299 = Autism and other psychoses with origin specific to childhood.
- 758.0 - 758.3 = Chromosomal Anomalies (includes Down's, Patau's and Edward's syndromes).
- 759.81 - 759.89 = Other and unspecified congenital anomalies (includes Fragile X and Prader-Willi syndromes).
- 760.71 = Alcohol affecting fetus or newborn via placenta or breast milk (Fetal Alcohol Syndrome (FAS)).
- ICD-10
- F70.0, F70.1, F70.8, F70.9 = Mild mental retardation;
- F71.0, F71.1, F71.8, F71.9 = Moderate mental retardation;
- F72.0, F72.1, F72.8, F72.9 = Severe mental retardation;
- F73.0, F73.1, F73.8, F73.9 = Profound mental retardation;
- F78.0, F78.1, F78.8, F78.9 = Other mental retardation;
- F79.0, F79.1, F79.8, F79.9 = Unspecified mental retardation;
- F84.0, F84.1, F84.3, F84.4, F84.5, F84.8, F84.9 = Pervasive developmental disorders;
- P04.3 = Fetus and newborn affected by maternal use of alcohol (this excludes fetal alcohol syndrome = Q86.0);
- Q86.0, Q86.1, Q86.2, Q86.8 = Congenital malformation syndromes due to known exogenous causes, not elsewhere classified;
- Q87.0, Q87.1, Q87.2, Q87.3, Q87.5, Q87.8 = Other specified congenital malformation syndromes affecting multiple systems;
- Q89.8 = Other specified congenital malformations;
- Q90.0, Q90.1, Q90.2, Q90.9 = Down's syndrome;
- Q91.0, Q91.1, 91.2, Q91.3, 91.4, Q91.5, 91.6, Q91.7 = Edward's syndrome and Patau's syndrome;
- Q93.0, Q93.1, Q93.2, Q93.3, Q93.4, Q93.5, Q93.6, Q93.7, Q93.8, Q93.9 = Monosomies and deletions from the autosomes, not elsewhere classified; and
- Q99.2 = Fragile X chromosome

10. Income [8,9]

The dissemination area-level after-tax income was obtained from the Postal Code Conversion File Plus Version 7A/7D for BC.[8] This variable is generated by Statistics Canada using administrative data sources and captures household size to generate a per-person equivalent measure.[2]

11. Vaccination status[10]

Not vaccinated: Individual has not received any vaccine dose by collection date.

Partially vaccinated: At least 14 days have passed since first dose inoculation and collection date.

Vaccinated: No less than 14 days have passed since second dose and collection date.[10]

References (definitions)

[1] Janjua NZ, Kuo M, Chong M, Yu A, Alvarez M, Cook D, et al. Assessing hepatitis C burden and treatment effectiveness through the British Columbia hepatitis testers cohort (BC-HTC): Design and characteristics of linked and unlinked participants. PLoS One 2016;11:e0150176. https://doi.org/10.1371/journal.pone.0150176.

[2] Brownell M, De Coster C, Penfold R, Derksen S, Au W, Schultz J, Dahl M. Manitoba Child Health Atlas Update. Winnipeg, MB: Manitoba Centre for Health Policy, 2008. Available at http://mchp-appserv.cpe.umanitoba.ca/concept/Codes_for_Delivery_Outcome_Baby_CHA_2008.pdf

[3] Quan H, Sundararajan V, Halfon P, Fong A, Burnand B, Luthi J-C, et al. Coding Algorithms for Defining Comorbidities in ICD-9-CM and ICD-10 Administrative Data. Med Care 2005;43:1130–9. https://doi.org/10.1097/01.mlr.0000182534.19832.83.

[4] Sundaram ME, Calzavara A, Mishra S, Kustra R, Chan AK, Hamilton MA, et al. Individual and social determinants of SARS-CoV-2 testing and positivity in Ontario, Canada: a population-wide study. CMAJ 2021;193:E723–34. https://doi.org/10.1503/CMAJ.202608.

[5] Janjua NZ, Islam N, Kuo M, et al. Identifying injection drug use and estimating population size of people who inject drugs using healthcare administrative datasets. Int J Drug Policy. 2018;55:31-39. https://doi:10.1016/j.drugpo.2018.02.001.

[6] Janjua NZ, Kuo M, Yu A, Alvarez M, Wong S, Cook D, et al. The Population Level Cascade of Care for Hepatitis C in British Columbia, Canada: The BC Hepatitis Testers Cohort (BC-HTC). EBioMedicine 2016;12:189–95. https://doi.org/10.1016/j.ebiom.2016.08.035.

[7] Algorithm developed and validated by BCCDC (BC Hepatitis Testers Cohort).

[8]Postal Code Conversion File Plus (PCCF+) Version 7A & 7D. Ottawa: Statistics Canada; 2016. Cat no 82F0086X. Available: https://www150.statcan.gc.ca/n1/en/catalogue/82F0086X (accessed 2022 May 13).

[9]2016 Census topic: income. Ottawa: Statistics Canada; 2017, updated 2019 Dec. 10. Available: https://www12.statcan.gc.ca/census-recensement/2016/rt-td/inc-rev-eng.cfm (accessed 2022 May 11).

[10]Fibke CD, Joffres Y, Tyson JR, Colijn C, Janjua NZ, Fjell C, et al. Spike Mutation Profiles Associated With SARS-CoV-2 Breakthrough Infections in Delta Emerging and Predominant Time Periods in British Columbia, Canada. Front Public Heal 2022;0:2038. https://doi.org/10.3389/FPUBH.2022.915363.

Section S3. Supplementary results

Table S2. Factors associated with COVID-19-related hospitalization in multivariable logistic regression* analysis among confirmed adult cases during 2021, ages 18-49, BC COVID-19 Cohort (n=115,874)

Parameter	Levels	AOR	LCI	UCI	p-value
**Sex** (ref. Female)	Male	1.05	0.97	1.14	0.257
**Asthma**		1.00	0.90	1.12	0.992
**Chronic obstructive pulmonary disease**		2.88	1.82	4.56	< .001
**Cirrhosis**		3.73	2.70	5.15	< .001
**Chronic kidney disease**		2.49	2.02	3.06	< .001
**Diabetes mellitus** (DM, ref. non-diabetic)	DM - Non-insulin dependent	2.22	1.82	2.70	< .001
	DM - Insulin dependent	3.85	2.97	4.99	< .001
**Heart failure**		2.11	1.36	3.29	< .001
**Hypertension**		1.79	1.53	2.10	< .001
**Injection drug use**		2.58	2.28	2.92	< .001
**Alcohol misuse**		1.62	1.42	1.84	< .001
**Immunosuppression**		2.08	1.74	2.48	< .001
**Alzheimer/dementia**		3.27	0.58	18.46	0.181
**Schizophrenia and psychotic disorders**		2.17	1.83	2.57	< .001
**Multiple sclerosis**		5.61	3.05	10.30	< .001
**Rheumatoid arthritis**		1.91	1.30	2.82	0.001
**Obesity**		1.88	1.57	2.26	< .001
**Weight loss**		1.47	1.10	1.96	0.009
**Intellectual and developmental disabilities**		2.10	1.33	3.31	0.002
**Cancer, lymphoma**		1.67	1.30	2.16	< .001
**Cancer, metastatic**		1.14	1.00	1.31	0.046
**Variant of concern** (ref. non-VOC)	Delta	2.86	2.37	3.46	< .001
	Alpha	1.66	1.34	2.06	< .001
	Beta	1.39	0.19	10.15	0.747
	Gamma	2.98	2.41	3.68	< .001
	Not sequenced	1.47	1.21	1.78	< .001
	Omicron	2.22	1.48	3.32	< .001
**Vaccination status** (ref. unvaccinated)	Partially vaccinated	0.49	0.43	0.56	< .001
	Vaccinated	0.15	0.13	0.18	< .001
**Income** (quintiles - ref. 1st)	2nd	0.87	0.77	0.97	0.014
	3rd	0.78	0.69	0.88	< .001
	4th	0.67	0.59	0.76	< .001

	5th	0.67	0.58	0.77	< .001
	Missing/unknown	0.66	0.55	0.79	< .001
**Health authority** (ref. Fraser)	Interior	1.05	0.93	1.17	0.454
	Northern	1.72	1.51	1.96	< .001
	Vancouver Coastal	0.81	0.72	0.90	< .001
	Vancouver Island	1.03	0.87	1.21	0.746
	Missing/unknown	0.55	0.20	1.48	0.235

AOR: adjusted odds ratio; LCI: lower confidence interval; UCI: upper confidence interval.

*Parkinsonism was removed from model due to zero counts.

Table S3. Factors associated with COVID-19-related hospitalization in multivariable logistic regression analysis among confirmed adult cases during 2021, ages 50-69, BC COVID-19 Cohort (n=37,435)

Parameter	Levels	AOR	LCI	UCI	p-value
**Sex** (ref. Female)	Male	1.58	1.45	1.71	< .001
**Asthma**		1.20	1.07	1.34	0.001
**Chronic obstructive pulmonary disease**		1.81	1.55	2.10	< .001
**Cirrhosis**		**2.55**	**1.96**	**3.31**	< .001
**Chronic kidney disease**		2.47	2.17	2.80	< .001
**Diabetes mellitus** (DM, ref. non-diabetic)	DM - Non-insulin dependent	1.40	1.25	1.56	< .001
	DM - Insulin dependent	3.24	2.75	3.82	< .001
**Heart failure**		1.50	1.21	1.86	< .001
**Hypertension**		1.36	1.24	1.48	< .001
**Injection drug use**		1.89	1.62	2.19	< .001
**Alcohol misuse**		1.36	1.17	1.57	< .001
**Immunosuppression**		1.89	1.59	2.26	< .001
**Alzheimer/dementia**		1.77	1.02	3.10	0.044
**Schizophrenia and psychotic disorders**		1.51	1.22	1.87	< .001
**Multiple sclerosis**		2.03	1.10	3.76	0.024
**Parkinsonism**		2.24	0.96	5.22	0.061
**Rheumatoid arthritis**		1.12	0.88	1.43	0.353
**Obesity**		1.72	1.46	2.02	< .001
**Weight loss**		1.44	1.17	1.77	< .001
**Intellectual and developmental disabilities**		2.25	1.44	3.52	< .001
**Cancer, lymphoma**		1.43	1.01	2.01	0.042
**Cancer, metastatic**		1.73	1.43	2.08	< .001
**Variant of concern** (ref. non-VO)	Delta	4.26	3.58	5.06	< .001
	Alpha	1.97	1.62	2.39	< .001
	Beta	0.93	0.20	4.31	0.923
	Gamma	3.97	3.27	4.83	< .001
	Not sequenced	1.38	1.16	1.65	< .001
	Omicron	2.55	1.66	3.90	< .001
**Vaccination status** (ref. unvaccinated)	Partially vaccinated	0.54	0.47	0.61	< .001
	Vaccinated	0.13	0.12	0.15	< .001

**Income** (quintiles - ref. 1st)	2nd	0.75	0.67	0.84	< .001
	3rd	0.73	0.65	0.82	< .001
	4th	0.64	0.56	0.72	< .001
	5th	0.57	0.50	0.65	< .001
	Missing/unknown	1.03	0.84	1.27	0.749
**Health authority** (ref. Fraser)	Interior	1.14	1.02	1.28	0.023
	Northern	1.70	1.50	1.94	< .001
	Vancouver Coastal	1.09	0.98	1.22	0.101
	Vancouver Island	1.40	1.20	1.63	< .001
	Missing/unknown	0.36	0.08	1.50	0.159

AOR: adjusted odds ratio; LCI: lower confidence interval; UCI: upper confidence interval.

Table S4. Factors associated with COVID-19-related hospitalization in multivariable logistic regression analysis among confirmed adult cases during 2021, ages ≥70, BC COVID-19 Cohort (n=9,200)

Parameter	Levels				
**Sex** (ref. Female)	Male	1.40	1.27	1.55	< .001
**Asthma**		1.04	0.90	1.19	0.618
**Chronic obstructive pulmonary disease**		1.73	1.49	2.01	< .001
**Cirrhosis**		**1.68**	**1.07**	**2.64**	0.023
**Chronic kidney disease**		1.85	1.65	2.07	< .001
**Diabetes mellitus** (DM, ref. non-diabetic)	DM - Non-insulin dependent	1.17	1.04	1.31	0.01
	DM - Insulin dependent	2.05	1.68	2.50	< .001
**Heart failure**		1.56	1.34	1.82	< .001
**Hypertension**		1.13	1.01	1.28	0.037
**Injection drug use**		0.85	0.46	1.58	0.612
**Alcohol misuse**		1.56	1.24	1.96	< .001
**Immunosuppression**		1.93	1.51	2.47	< .001
**Alzheimer/dementia**		1.74	1.33	2.28	< .001
**Schizophrenia and psychotic disorders**		1.89	1.32	2.71	< .001
**Multiple sclerosis**		2.12	0.82	5.45	0.12
**Parkinsonism**		2.31	1.46	3.65	< .001
**Rheumatoid arthritis**		1.55	1.23	1.96	< .001
**Obesity**		1.08	0.81	1.43	0.602
**Weight loss**		1.57	1.27	1.93	< .001
**Intellectual and developmental disabilities**		1.33	0.58	3.05	0.5
**Cancer, lymphoma**		1.56	1.16	2.09	0.004
**Cancer, metastatic**		1.30	1.06	1.59	0.011
**Variant of concern** (ref. non-VOC)	Delta	2.65	2.16	3.26	< .001
	Alpha	1.11	0.88	1.40	0.369
	Beta	1.62	0.45	5.85	0.459
	Gamma	1.93	1.50	2.49	< .001
	Not sequenced	0.92	0.76	1.11	0.384

	Omicron	2.20	1.41	3.43	< .001
**Vaccination status** (ref. unvaccinated)	Partially vaccinated	0.64	0.56	0.74	< .001
	Vaccinated	0.20	0.17	0.23	< .001
**Income** (quintiles - ref. 1st)	2nd	0.74	0.64	0.85	< .001
	3rd	0.69	0.60	0.80	< .001
	4th	0.70	0.60	0.83	< .001
	5th	0.61	0.52	0.72	< .001
	Missing/unknown	0.97	0.71	1.32	0.847
**Health authority** (ref. Fraser)	Interior	1.29	1.12	1.47	< .001
	Northern	1.54	1.28	1.84	< .001
	Vancouver Coastal	1.25	1.09	1.45	0.002
	Vancouver Island	0.96	0.77	1.18	0.675
	Missing/unknown	0.39	0.11	1.40	0.148

Table S5. Factors associated with COVID-19-related ICU admission in multivariable logistic regression* analysis among confirmed adult hospitalization cases during 2021, ages 18-49, BC COVID-19 Cohort (n=2,691)

Parameter	Levels	AOR	LCI	UCI	p-value
**Sex** (ref. Female)	Male	1.60	1.33	1.94	< .001
**Asthma**		1.10	0.86	1.41	0.446
**Chronic obstructive pulmonary disease**		0.99	0.41	2.38	0.977
**Cirrhosis**		**2.90**	**1.65**	**5.09**	< .001
**Chronic kidney disease**		1.83	1.22	2.75	0.004
**Diabetes mellitus** (DM, ref. non-diabetic)	DM - Non-insulin dependent	1.50	1.02	2.21	0.04
	DM - Insulin dependent	1.49	0.92	2.44	0.107
**Heart failure**		2.01	0.91	4.45	0.085
**Hypertension**		1.17	0.85	1.61	0.35
**Injection drug use**		0.86	0.65	1.14	0.285
**Alcohol misuse**		1.31	0.98	1.74	0.067
**Immunosuppression**		0.85	0.56	1.27	0.423
**Schizophrenia and psychotic disorders**		0.51	0.34	0.76	0.001
**Multiple sclerosis**		1.31	0.34	5.08	0.694
**Rheumatoid arthritis**		2.18	1.01	4.71	0.047
**Obesity**		2.15	1.49	3.10	< .001
**Weight loss**		0.95	0.52	1.72	0.863
**Intellectual and developmental disabilities**		1.19	0.64	2.21	0.589
**Cancer, lymphoma**		0.92	0.32	2.66	0.884
**Cancer, metastatic**		0.89	0.51	1.58	0.697
**Variant of concern** (ref. non-VOC)	Delta	1.51	0.97	2.35	0.068
	Alpha|Beta**	1.18	0.71	1.96	0.521
	Gamma	1.84	1.13	2.99	0.014
	Not sequenced	1.08	0.69	1.70	0.734
	Omicron	0.26	0.03	2.07	0.203
**Vaccination status** (ref. unvaccinated)	Partially vaccinated	0.50	0.35	0.72	< .001

	Vaccinated	0.35	0.20	0.61	< .001
**Income** (quintiles - ref. 1st)	2nd	1.11	0.85	1.44	0.433
	3rd	1.03	0.78	1.37	0.81
	4th	1.04	0.78	1.40	0.774
	5th	0.94	0.68	1.29	0.695
	Missing/unknown	1.08	0.71	1.63	0.731
**Health authority** (ref. Fraser)	Interior	1.29	0.99	1.67	0.06
	Northern	1.27	0.95	1.69	0.107
	Vancouver Coastal	1.13	0.87	1.48	0.36
	Vancouver Island	1.28	0.87	1.89	0.204
	Missing/unknown	1.41	0.13	14.69	0.776

AOR: adjusted odds ratio; LCI: lower confidence interval; UCI: upper confidence interval.

*Alzheimer/dementia and Parkinsonism were removed from model due to zero counts.

**VOC: Alpha and Beta were collapsed together due to low counts.

Table S6. Factors associated with COVID-19-related ICU admission in multivariable logistic regression analysis among confirmed adult hospitalization cases during 2021, ages 50-69, BC COVID-19 Cohort (n=3,273)

Parameter	Levels	AOR	LCI	UCI	p-value
**Sex** (ref. Female)	Male	1.3	1.11	1.52	0.001
**Asthma**		0.99	0.81	1.22	0.931
**Chronic obstructive pulmonary disease**		1.33	1.02	1.74	0.032
**Cirrhosis**		**1.73**	**1.16**	**2.58**	0.007
**Chronic kidney disease**		1.19	0.95	1.48	0.124
**Diabetes mellitus** (DM, ref. non-diabetic)	DM - Non-insulin dependent	1.27	1.04	1.55	0.02
	DM - Insulin dependent	1.64	1.26	2.14	< .001
**Heart failure**		1.16	0.82	1.63	0.412
**Hypertension**		0.98	0.83	1.15	0.777
**Injection drug use**		0.79	0.6	1.04	0.09
**Alcohol misuse**		0.94	0.72	1.23	0.653
**Immunosuppression**		1.52	1.13	2.05	0.006
**Alzheimer/dementia**		0.69	0.23	2.04	0.506
**Schizophrenia and psychotic disorders**		0.6	0.41	0.9	0.013
**Multiple sclerosis**		0.18	0.02	1.46	0.108
**Parkinsonism**		0.42	0.05	3.79	0.441
**Rheumatoid arthritis**		1.01	0.64	1.6	0.951
**Obesity**		1.75	1.32	2.32	< .001
**Weight loss**		0.9	0.62	1.3	0.559
**Intellectual and developmental disabilities**		0.89	0.39	2.03	0.79
**Cancer, lymphoma**		1.38	0.77	2.46	0.283
**Cancer, metastatic**		0.93	0.66	1.31	0.658
**Variant of concern** (ref. non-VOC)	Delta	1.73	1.22	2.45	0.002
	Alpha|Beta*	1.24	0.84	1.83	0.274

	Gamma	1.58	1.07	2.32	0.02
	Not sequenced	1.21	0.85	1.72	0.284
	Omicron	1.56	0.62	3.94	0.347
**Vaccination status** (ref. unvaccinated)	Partially vaccinated	0.73	0.58	0.93	0.012
	Vaccinated	0.4	0.29	0.54	< .001
**Income** (quintiles - ref. 1st)	2nd	1.09	0.88	1.35	0.415
	3rd	1.06	0.85	1.33	0.583
	4th	1.09	0.86	1.38	0.468
	5th	0.86	0.67	1.12	0.267
	Missing/unknown	1.19	0.8	1.78	0.38
**Health authority** (ref. Fraser)	Interior	1.08	0.87	1.33	0.5
	Northern	0.92	0.72	1.18	0.506
	Vancouver Coastal	0.91	0.74	1.13	0.409
	Vancouver Island	1.26	0.94	1.69	0.127
	Missing/unknown**	NA	NA	NA	0.961

AOR: adjusted odds ratio; LCI: lower confidence interval; UCI: upper confidence interval.

*VOC: Alpha and Beta were collapsed together due to low counts.

**Undetermined due to low cell counts

Table S7. Factors associated with COVID-19-related ICU admission in multivariable logistic regression analysis among confirmed adult hospitalization cases during 2021, ages ≥70, BC COVID-19 Cohort (n=2,582)

Parameter	Levels	AOR	LCI	UCI	p-value
**Sex** (ref. Female)	Male	1.40	1.16	1.69	< .001
**Asthma**		1.07	0.83	1.37	0.627
**Chronic obstructive pulmonary disease**		1.08	0.83	1.39	0.578
**Cirrhosis**		**1.72**	**0.91**	**3.27**	0.096
**Chronic kidney disease**		0.98	0.79	1.20	0.813
**Diabetes mellitus** (DM, ref. non-diabetic)	DM - Non-insulin dependent	1.05	0.85	1.31	0.642
	DM - Insulin dependent	1.44	1.05	1.97	0.024
**Heart failure**		0.74	0.56	0.96	0.025
**Hypertension**		0.71	0.57	0.88	0.002
**Injection drug use**		2.15	0.86	5.37	0.103
**Alcohol misuse**		0.67	0.45	1.01	0.058
**Immunosuppression**		1.58	1.09	2.28	0.015
**Alzheimer/dementia**		0.31	0.16	0.60	< .001
**Schizophrenia and psychotic disorders**		0.64	0.33	1.23	0.179
**Multiple sclerosis**		1.25	0.30	5.22	0.76
**Parkinsonism**		0.55	0.21	1.44	0.224
**Rheumatoid arthritis**		0.79	0.51	1.22	0.284
**Obesity**		2.18	1.40	3.40	< .001
**Weight loss**		0.50	0.34	0.76	< .001
**Intellectual and developmental disabilities**		0.72	0.14	3.67	0.691

**Cancer, lymphoma**		1.21	0.76	1.94	0.423
**Cancer, metastatic**		1.18	0.85	1.65	0.328
**Variant of concern** (ref. non-VOC)	Delta	0.86	0.60	1.23	0.409
	Alpha|Beta*	0.25	0.78	1.80	0.418
	Gamma	1.07	0.69	1.66	0.768
	Not sequenced	0.98	0.69	1.38	0.895
	Omicron	0.64	0.23	1.83	0.41
**Vaccination status** (ref. unvaccinated)	Partially vaccinated	0.59	0.46	0.78	< .001
	Vaccinated	0.58	0.43	0.78	< .001
**Income** (quintiles - ref. 1st)	2nd	0.89	0.69	1.15	0.384
	3rd	0.70	0.52	0.92	0.012
	4th	1.02	0.77	1.36	0.89
	5th	0.90	0.66	1.23	0.517
	Missing/unknown	1.09	0.64	1.87	0.744
**Health authority** (ref. Fraser)	Interior	1.28	1.00	1.65	0.051
	Northern	1.42	1.04	1.93	0.027
	Vancouver Coastal	1.14	0.87	1.48	0.344
	Vancouver Island	1.24	0.83	1.85	0.297
	Missing/unknown**	NA	NA	NA	0.967

AOR: adjusted odds ratio; LCI: lower confidence interval; UCI: upper confidence interval.

*VOC: Alpha and Beta were collapsed together due to low counts.

**Undetermined due to low cell counts
